# Introducing tumor necrosis factor as a prominent player in celiac disease and type 1 diabetes mellitus 

**Published:** 2019

**Authors:** Sina Rezaei-Tavirani, Mohammad Rostami-Nejad, Reza Vafaee, Ensieh Khalkhal, Aliasghar Keramatinia, Mohammad Javad Ehsani-Ardakani, Mohammadreza Razzaghi

**Affiliations:** 1 *Basic and Molecular Epidemiology of Gastrointestinal Disorders Research Center, Research Institute for Gastroenterology and Liver Diseases, Shahid Beheshti University of Medical Sciences, Tehran, Iran*; 2 *Gastroenterology and Liver Diseases Research Center, Research Institute for Gastroenterology and Liver Diseases, Shahid Beheshti University of Medical Sciences, Tehran, Iran*; 3 *Proteomics Research Center, Shahid Beheshti University of Medical Sciences, Tehran, Iran*; 4 *Proteomics Research Center, Faculty of Paramedical Sciences, Shahid Beheshti University of Medical Sciences, Tehran, Iran*; 5 *Faculty of Medicine, Shahid Beheshti University of Medical Sciences, Tehran, Iran*; 6 *Laser Application in Medical Sciences Research Center, Shahid Beheshti University of Medical Sciences, Tehran, Iran*

**Keywords:** Celiac disease, Type 1 diabetes mellitus, TNF, Insulin

## Abstract

**Aim:**

This study aimed to screen the common genes between celiac disease (CD) and type 1 diabetes mellitus to find critical ones.

**Background:**

Celiac disease is a chronic autoimmune disorder which is correlated to type 1 diabetes mellitus (T1DM) in several molecular pathways. Understanding the clear common molecular mechanism of both diseases is of interest to scientists.

**Methods:**

The related genes to the CD and T1DM were obtained from disease query of STRING and included in two separated PPI networks by Cytoscape software version 3.7.1. The networks were analyzed by network analyzer and the hub nodes were determined. The common hubs between the two networks were selected for further analysis and enriched via gene ontology using ClueGO plugin of Cytoscape software. Also, an action map was provided by Cluepedia application of Cytoscape software.

**Results:**

Two separated networks of 2000 and 430 genes were constructed related to T1DM and CD, respectively. A total of 84 and 28 hubs were determined for T1DM and CD, respectively. There were 11 common hubs between the two networks. The first top hubs of Type 1 Diabetes Mellitus and CD networks were insulin (INS) and tumor necrosis factor (TNF), respectively. Also, 77 biological terms and pathways (in five clusters) were related to the common hubs. Action map revealed a close relationship between hubs.

**Conclusion:**

The result of this study indicated that TNF is key mediator of immune reactions in celiac disease and type 1 diabetes mellitus.

## Introduction

Celiac disease (CD) is a chronic autoimmune disorder in genetically susceptible individuals which is triggered by wheat gluten. It is a multifactorial disease induced by genetic (HLA-DQ2 or HLA-DQ8) and environmental factors (gluten) (1, 2). CD is associated with some diseases including Type 1 Diabetes Mellitus (T1DM) (3-5). T1DM is characterized by decreased endogenous insulin due to autoimmune destruction of beta cells of pancreatic islets. Blood glucose levels can no longer be maintained in a physiologic range without exogenous insulin (6). It has been shown that autoimmune diseases such as CD are more prevalent among T1DM patients (7, 8); thus, patients with T1DM are at high risk of developing CD. The estimated prevalence of CD in patients with T1DM is approximately between 3 and 16% with a mean prevalence of 8% (9-11). It has prompted the recommendation to screen T1DM patients for CD at diagnosis then annually for the first 4 years, once every 2 years, and the following for 6 years (12, 13). CD and T1DM, immune-mediated diseases with clinical and pathogenic overlap, share a number of common risk factors including genetics, environment, and immune dysregulation. Both diseases are inherited diseases with strong genetic components notably HLA. HLA-DQ2 is found in about 90% of CD and 55% of T1DM patients, while HLA-DQ8 is found in about 10% of CD and 70% of T1DM patients (14). There are also several non-HLA loci that overlap between T1DM and CD (15). The overlap of genetic variants between T1DM and CD (including HLA and non-HLA) emphasizes common pathogenic mechanisms and explains increased prevalence of concomitant disease. There are several common environmental risk factors that increase the risk of developing T1DM or CD autoimmunity factors including introduction of cereals, infant feeding practices, and lack of breast-feeding, with the gut microbiome and viral infections appearing to contributed to the risk of developing both CD and T1DM individually (16-18). 

Gene and environmental interactions which are susceptible to the development of both T1DM and CD have not yet been established. In this study, common features of both diseases were investigated by network analysis to find critical elements and pathways in development of CD and diabetes. 

## Methods

Since there are many genes related to several diseases in STRING database, the related genes to the celiac and T1DM were obtained from a disease query of STRING. Two separated PPI networks were constructed for both celiac and T1DM diseases by Cytoscape software version 3.7.1 via undirected connections (19). Network analyzer was applied to analyze the network and identify the central nodes. Hubs of the two networks were determined based on degree value. The mean value of degree + 2 standard deviation (SD) was considered as cutoff value (20). The top nodes which were characterized by degree value above cutoff were identified as hubs. 

The common hubs between the two networks were selected for further analysis. Description of the common hubs was downloaded from STRING database and summarized. The connection between the common hubs was visualized as a subnetwork. 

**Figure 1:**
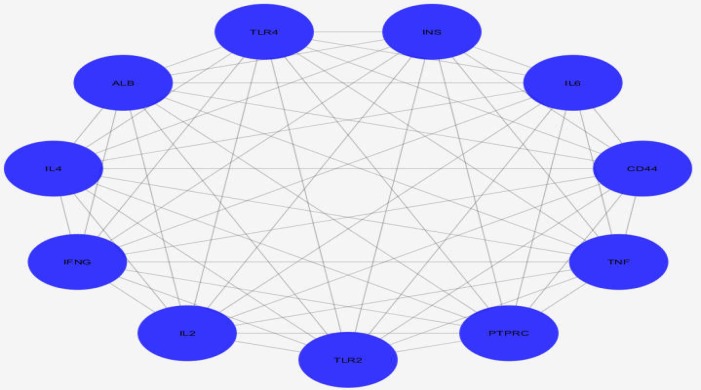
A subnetwork including the common hubs of the CD and Type 1 Diabetes Mellitus networks and their undirected connections

**Figure 2:**
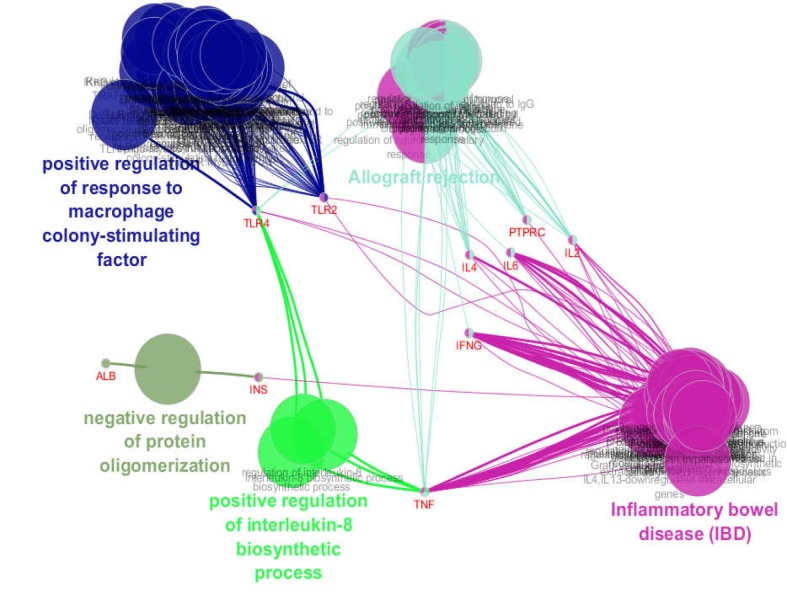
A total of 77 biological terms and pathways which are clustered in 5 groups (Kappa score = 3) in relationship with the common hub nodes are represented. The name of cluster and also protein are hid lighted while the other terms are background

**Figure 3:**
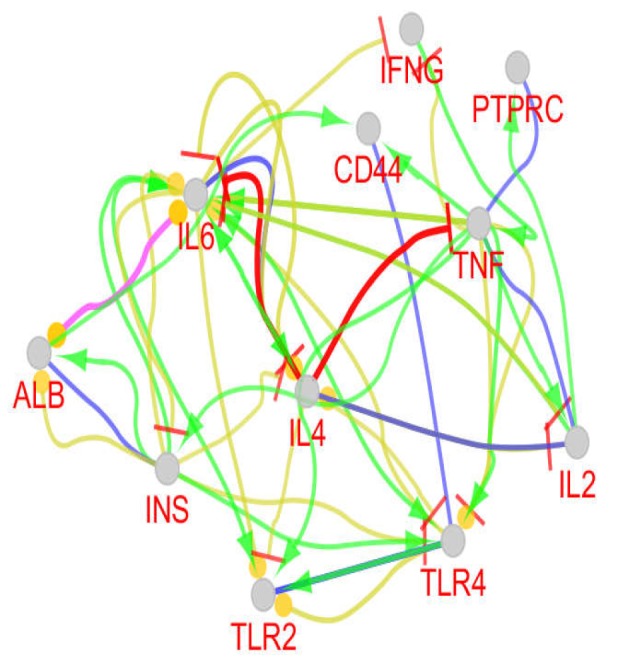
The action map of the common hub nodes; Pink, yellow, red, green, blue, and black colors represent catalysis, expression, inhibition, activation, binding, and reaction, respectively

Gene ontology enrichment for the common hubs was done via CluGO plugin of Cytoscape software. The significant biological terms based on P-value ≤ 0.05 were identified. The terms were clustered and the clusters were connected to the relevant hubs. 

The action map of common hub genes including binding, expression, inhibition, activation, catalysis, and reaction were provided by Clupedia application of Cytoscape software. 

## Results

A total of 2000 genes related to the T1DM and 430 genes related to CD were obtained from disease query of STRING database. The two networks were constructed and analyzed (the data are not shown). The mean and SD of degree value in T1DM network were 72 and 82 respectively. Thus, the nodes characterized by degree value above 236 were introduced as hubs of T1DM. The T1DM and CD network were characterized by 84 and 28 hubs respectively. These parameters for CD network were determined as 30 and 34.5 respectively. In this regard, the degree value of 99 as cutoff was considered to introduce hubs of CD network. 

As observed in Table 1, 11 common hubs were introduced for the two networks. Descriptions of the common hubs from STRING database were downloaded and summarized in Table 1. As presented in Table 1, the first top hubs of T1DM and CD networks are INS and TNF respectively. However, INS as a common hub is ranked as the fourth top hub related to celiac disease. Also, TNF is the fourth top hub related to the diabetes. As displayed in Figure 1, all common hubs are connected to each other. 

A total of 77 biological terms and pathways which are related to the common hubs were obtained from KEGG, REACTOME_Reactions, REACTOME_ Pathways, GO_Cellular Component-EBI-UniProt-GOA, and GO_Biological Process-EBI-UniProt-GOA (updated at 27.02.2019). The terms and pathways based on Kappa score = 3 are clustered in 5 groups. These terms and pathways in relationship with the related common hubs are shown in Figure 2. In Figure 3, the action map of the common hub nodes including binding, expression, inhibition, activation, catalysis, and reaction is illustrated. In the action map, 11 common nodes are connected to each other via non-homogenous edges signaling the different types of relationships between them.

**Table 1:** The common hub nodes between CD and T1DM networks; Descriptions have been downloaded from the STRING database and summarized. D_1 _and D_2_ refer to the degree values of the nodes in the CD and T1DM respectively

R	Name	Description	D_1_	D_2_
1	TNF	Tumor necrosis factor superfamily mainly secreted by macrophages and can induce cell death of certain tumor cell lines. It is potent pyrogen causing fever and is implicated in the induction of cachexia, stimulating cell proliferation, and cell differentiation. Impairs regulatory T-cells (Treg) function in individuals with rheumatoid arthritis. It is key mediator of cell death in the anticancer action of BCG-stimulated neutrophils in combination with DIABLO/SMAC mimetic in the RT4v6 bladder cancer cell line.	172	595
2	IL6	B-cell stimulatory factor 2; Cytokine with a wide variety of biological functions. It is a potent inducer of the acute phase response. Plays an essential role in the final differentiation of B-cells into Ig- secreting cells involved in lymphocyte and monocyte differentiation. Acts on B-cells, T-cells, hepatocytes, hematopoietic progenitor cells and cells of the CNS. Required for the generation of T(H)17 cells. Also acts as a myokine. It is discharged into the bloodstream after muscle contraction and acts to increase the breakdown of fats and to improve insulin resistance. It induces myeloma and plasmacytoma growth and induces nerve cells differentiation as an interferon.	166	672
3	IL2	T-cell growth factor; Produced by T-cells in response to antigenic or mitogenic stimulation, this protein is required for T-cell proliferation and other activities crucial to regulation of the immune response. Can stimulate B-cells, monocytes, lymphokine- activated killer cells, natural killer cells, and glioma cells; Interleukins	159	319
4	INS	Insulin; Insulin decreases blood glucose concentration. It increases cell permeability to monosaccharides, amino acids and fatty acids. It accelerates glycolysis, the pentose phosphate cycle, and glycogen synthesis in liver.	147	1002
5	IL4	Lymphocyte stimulatory factor 1; Participates in at least several B-cell activation processes as well as of other cell types. It is a costimulator of DNA-synthesis. It induces the expression of class II MHC molecules on resting B-cells. It enhances both secretion and cell surface expression of IgE and IgG1. It also regulates the expression of the low affinity Fc receptor for IgE (CD23) on both lymphocytes and monocytes. Positively regulates IL31RA expression in macrophages (By similarity); Interleukins	135	326
6	ALB	Serum albumin; Serum albumin, the main protein of plasma, has a good binding capacity for water, Ca(2+), Na(+), K(+), fatty acids, hormones, bilirubin and drugs. Its main function is the regulation of the colloidal osmotic pressure of blood. Major zinc transporter in plasma, typically binds about 80% of all plasma zinc; Belongs to the ALB/AFP/VDB family.	132	727
7	PTPRC	Protein tyrosine phosphatase, receptor type, C; Protein tyrosine-protein phosphatase required for T-cell activation through the antigen receptor. Acts as a positive regulator of T-cell coactivation upon binding to DPP4. The first PTPase domain has enzymatic activity, while the second one seems to affect the substrate specificity of the first one. Upon T-cell activation, recruits and dephosphorylates SKAP1 and FYN. Dephosphorylates LYN, and thereby modulates LYN activity (By similarity); Belongs to the protein-tyrosine phosphatase family. Receptor class 1/6 subfamily.	126	296
8	IFNG	Immune interferon; Produced by lymphocytes activated by specific antigens or mitogens. IFN-gamma, in addition to having antiviral activity, has important immunoregulatory functions. It is a potent activator of macrophages, it has antiproliferative effects on transformed cells and it can potentiate the antiviral and antitumor effects of the type I interferons; Belongs to the type II (or gamma) interferon family.	122	244
9	TLR4	Toll-like receptor 4; Cooperates with LY96 and CD14 to mediate the innate immune response to bacterial lipopolysaccharide (LPS). Acts via MYD88, TIRAP and TRAF6, leading to NF-kappa-B activation, cytokine secretion and the inflammatory response. Also involved in LPS-independent inflammatory responses triggered by free fatty acids, such as palmitate, and Ni(2+). Responses triggered by Ni(2+) require non-conserved histidines and are, therefore, species-specific. Both M.tuberculosis HSP70 (dnaK) and HSP65 (groEL-2) act via this protein to stimulate NF-kappa-B expression. In complex with TLR6, promotes sterile inflammation in monocytes/macrophages in response to oxidized low-density lipoprotein (oxLDL) or amyloid-beta 42. In this context, the initial signal is provided by oxLDL- or amyloid-beta 42-binding to CD36. This event induces the formation of a heterodimer of TLR4 and TLR6, which is rapidly internalized and triggers inflammatory response, leading to the NF-kappa-B-dependent production of CXCL1, CXCL2 and CCL9 cytokines, via MYD88 signaling pathway, and CCL5 cytokine, via TICAM1 signaling pathway, as well as IL1B secretion. Binds electronegative LDL (LDL(-)) and mediates the cytokine release induced by LDL(-). Stimulation of monocytes in vitro with M.tuberculosis PstS1 induces p38 MAPK and ERK1/2 activation primarily via TLR2, but also partially via this receptor.	112	360
10	CD44	GP90 lymphocyte homing/adhesion receptor; Receptor for hyaluronic acid (HA). Mediates cell-cell and cell-matrix interactions through its affinity for HA, and possibly also through its affinity for other ligands such as osteopontin, collagens, and matrix metalloproteinases (MMPs). Adhesion with HA plays an important role in cell migration, tumor growth and progression. In cancer cells, may play an important role in invadopodia formation. Also involved in lymphocyte activation, recirculation and homing, and in hematopoiesis. Altered expression or dysfunction causes numerous pathogenic phenotypes. Great protein heterogeneity due to numerous alternative splicing and post-translational modification events. Receptor for LGALS9; the interaction enhances binding of SMAD3 to the FOXP3 promoter, leading to up-regulation of FOXP3 expression and increased induced regulatory T (iTreg) cell stability and suppressive function (By similarity); Blood group antigens	103	295
11	TLR2	Toll/interleukin-1 receptor-like protein 4; Cooperates with LY96 to mediate the innate immune response to bacterial lipoproteins and other microbial cell wall components. Cooperates with TLR1 or TLR6 to mediate the innate immune response to bacterial lipoproteins or lipopeptides. Acts via MYD88 and TRAF6, leading to NF-kappa-B activation, cytokine secretion and the inflammatory response. May also activate immune cells and promote apoptosis in response to the lipid moiety of lipoproteins. Acts as a receptor for M.tuberculosis lipoproteins LprA, LprG, LpqH and PstS1, some lipoproteins are dependent on other coreceptors (TLR1, CD14 and/or CD36); the lipoproteins act as agonists to modulate antigen presenting cell functions in response to the pathogen. M.tuberculosis HSP70 (dnaK) but not HSP65 (groEL-2) acts via this protein to stimulate NF-kappa-B expression. Recognizes M.tuberculosis major T-antigen EsxA (ESAT-6) which inhibits downstream MYD88-dependent signaling (shown in mouse) (By similarity). Forms activation clusters composed of several receptors depending on the ligand, these clusters trigger signaling from the cell surface and subsequently are targeted to the Golgi in a lipid-raft dependent pathway	101	279

## Discussion

It is clear that T1DM is a known disorder associated with CD. There are 3,260,000 documents about T1DM when “diabetes” is searched in google scholar search Engine (https://scholar.google.com/scholar?hl=en&as_sdt=0%2C5&q=diabetes&oq=diab). In a similar search, 404,000 documents about CD with “celiac” keyword were obtained (https://scholar.google.com/scholar?hl= en&as_sdt=0%2C5&q=celiac&btnG=). Thus, it is expected that there are more genes related to T1DM than CD in STRING database. In this study, 2000 proteins associated with T1DM were found but only 430 ones were obtained for CD. In this regard, it can be concluded that the network analysis leads to introduction of more numbers of hubs for T1DM versus CD. Our results indicated that there are 84 and 28 hubs for T1DM and CD networks respectively.

As presented in Table 1, about 40% of CD hubs (11 genes) are common between both diseases. Various functions are attributed to the common hub genes, but immunological activities are prominent tasks that are highlighted in Table 1 . This finding matches the nature of both T1DM and celiac disease. As illustrated in Figure 1, the common hubs are connected to each other and interact equally in a subnetwork. It is a presentation of a related functions which are controlled by the central genes.

 Gene ontology findings revealed that there are 77 biological terms relevant to the hub nodes (see Figure 2). The terms are clustered in five groups; the smallest cluster is “negative regulation of protein oligomerization”. This term is connected to the ALB and INS. Since INS is the first ranked hub in T1DM as well as the fourth hub in CD, it seems that negative regulation of protein oligomerization is an important cluster which is involved in both diseases. Importance of insulin signaling which was related to protein oligomerization in T1DM has been reported by TR Bomfin et al. (21). As observed in Figure 2, INS is connected to the other important cluster, “Inflammatory bowel disease (IBD)”. IBD is a chronic disorder characterized by abdominal pain, diarrhea, and gastrointestinal bleeding (22). Investigations have indicated that there is a significant correlation between CD and IBD (23). The important role of INS as a common hub in T1DM and CD is confirmed via involvement of INS in the protein oligomerization and IBD clusters. 

TNF, the top node of CD network, is connected directly to the “IBD”, “positive regulation of interleukin-8 biosynthetic process”, and “allograft” rejection clusters. This hub is also linked indirectly to the other two clusters. The role of TNF in T1DM has been confirmed by DE Moller et al. (24). As presented in Figure 3, TNF activates INS, IL2, IL6, CD44, TLR2, and TLR4 directly. Since INS, IL6, and IL2 activate ALB, IL4, and PTPRC respectively, it can be concluded that TNF activates all hub genes (except IFNG) directly and indirectly. Activation is the noticeable action in Figure 3. Since innate immune activation is a significant characterization of CD (25, 26) and Type 2 Diabetes Mellitus is a well-known disease tied to the activation of immune system (27), the finding emphasizes the notable role of TNF.

IL6 is the second and third hub in the CD and T1DM network respectively. This central gene is connected to all hubs (except PTPRC) via 17 connections in the action map. It is related to most hub genes via expression correlation links. This finding supports the fact that not only control of immune system function but also gene expression pattern is affected in both diseases. These findings are consistent with previous investigations about CD and T1DM (28-30). 

There are several pieces of evidence about significant roles of the other hubs in promotion of CD and diabetes. TLRs polymorphism is reported frequently in CD and T1DM (31-33). Serum level changes of ALB in both diseases have been reported by several investigators (34, 35). It can be concluded that the introduced 11 central genes are a suitable gene set which clear common pathways between the two studied diseases.

The findings indicate that the part of immune system that is controlled mainly by TNF is the common feature in CD and diabetes. This prevailing role of TNF warrants more investigation about the related pathways to control diseases in patients.
